# Decreased Protein C Pathway Activity in COVID-19 Compared to Non-COVID Sepsis: An Observational and Comparative Cohort Study

**DOI:** 10.3390/biomedicines12091982

**Published:** 2024-09-02

**Authors:** Heiko Rühl, Christian Bode, Tobias Becher, Sebastian Eckert, Ghaith Mohsen, Hannah L. McRae, Jens Müller, Sara Reda, Dirk Loßnitzer, Johannes Oldenburg, Christian Putensen, Bernd Pötzsch

**Affiliations:** 1 Institute of Experimental Hematology and Transfusion Medicine, University Hospital Bonn, 53127 Bonn, Germany; sebastian.eckert@ukbonn.de (S.E.); hannah.mcrae@ukbonn.de (H.L.M.); jens.mueller@ukbonn.de (J.M.); sara.reda1@uk-koeln.de (S.R.); johannes.oldenburg@ukbonn.de (J.O.); b.poetzsch@ukbonn.de (B.P.); 2 Department of Anesthesiology and Intensive Care Medicine, University Hospital Bonn, 53127 Bonn, Germany; christian.bode@ukbonn.de (C.B.); ghaith.mohsen@ukbonn.de (G.M.); christian.putensen@ukbonn.de (C.P.); 3 First Department of Medicine, University Medical Centre Mannheim, Faculty of Medicine Mannheim, University of Heidelberg, 68167 Mannheim, Germany; tobiasbecher@gmx.net; 4 Department of Cardiology, Angiology and Pulmonology, University Hospital Heidelberg, 69120 Heidelberg, Germany; diloss@web.de

**Keywords:** COVID-19, protein C, sepsis, thrombin, thrombophilia

## Abstract

Sepsis-associated coagulopathy increases risk of mortality. Impairment of the anticoagulant protein C (PC) pathway may contribute to the thrombotic phenotype in coronavirus disease 2019 (COVID-19) sepsis. This study assessed the functionality of this pathway in COVID-19 and non-COVID sepsis by measuring its key enzymes, thrombin and activated PC (APC). The study population included 30 patients with COVID-19, 47 patients with non-COVID sepsis, and 40 healthy controls. In healthy controls, coagulation activation and subsequent APC formation was induced by 15 µg/kg recombinant activated factor VII one hour before blood sampling. APC and thrombin in plasma were measured using oligonucleotide-based enzyme capture assays. The indirect thrombin markers prothrombin-fragment 1+2 (F1+2) and thrombin-antithrombin complex (TAT) were also measured. Compared with stimulated healthy controls, median thrombin, F1+2, and TAT levels were higher in patients with COVID-19 (up to 6-fold, *p* < 2 × 10^−6^) and non-COVID sepsis (up to 4.7-fold, *p* < 0.010). APC levels were 2.4-fold higher in patients with COVID-19 (7.44 pmol/L, *p* = 0.011) and 3.4-fold higher in non-COVID sepsis patients (10.45 pmol/L, *p* = 2 × 10^−4^) than in controls (3.08 pmol/L). Thrombin markers and APC showed correlation in both COVID-19 (r = 0.364–0.661) and non-COVID sepsis patients (r = 0.535–0.711). After adjustment for PC levels, median APC/thrombin, APC/F1+2, and APC/TAT ratios were 2-fold (*p* = 0.036), 6-fold (*p* = 3 × 10^−7^) and 3-fold (*p* = 8 × 10^−4^) lower in the COVID-19 group than in the non-COVID sepsis group, and the latter two were also lower in the COVID-19 group than in stimulated healthy controls. In conclusion, it was found that a comparatively lower anticoagulant APC response in COVID-19 patients as compared to non-COVID sepsis patients, potentially linked to endothelial dysfunction, contributes to the prothrombotic phenotype of COVID-19 sepsis.

## 1. Introduction

Sepsis, a dysregulated host response to infection characterized by organ failure, is commonly associated with coagulation abnormalities [1,2,3]. The extent of hemostatic alterations in sepsis ranges from subclinical coagulation activation to fulminant disseminated intravascular coagulation. Disseminated intravascular coagulation is characterized by microvascular thrombosis and concomitant severe hemorrhage [4]. Pathomechanisms causing a prothrombotic state in sepsis include tissue factor-induced coagulation activation, platelet activation, and downregulation of fibrinolysis, along with disorders of anticoagulant pathways like the protein C (PC) system. Hemorrhage, which can be the dominant presentation of coagulopathy in sepsis, is caused by the consumption of coagulation factors and platelets [2,3,4,5]. Critically ill patients with coronavirus disease 2019 (COVID-19) fulfill the sepsis criteria, but COVID-19 and non-COVID sepsis are associated with different plasma coagulation abnormalities, such as systemic thrombotic complications, which are particularly pronounced with COVID-19 [6,7].

The key component of the PC system is the protein of the same name, which binds to the endothelial PC receptor (EPCR) and is then activated by the thrombin–thrombomodulin (TM) complex [8,9]. Activated PC (APC) exerts anticoagulant effects through protein S-enhanced inactivation of the activated coagulation factors V and VIII, and anti-inflammatory and antiapoptotic effects through protease activated receptors (PAR) and EPCR [10]. In non-COVID sepsis, the PC system is impaired by reduced plasma levels of PC and protein S, and downregulation of EPCR and TM [2]. Indirect evidence that the PC system may be affected by COVID-19 comes from low PC levels in hospitalized patients with COVID-19, which have been associated with increased mortality and increased levels of soluble TM and EPCR [5,11,12,13,14,15]. In addition, there is evidence from autopsy data of endotheliopathies and downregulation of TM and EPCR in the lungs of COVID-19 patients [16].

The aim of this study was to assess the functionality of the PC pathway in COVID-19 and non-COVID sepsis patients. Based on the available evidence, we hypothesized that the extent of APC formation in response to thrombin generation would differ between these types of sepsis. We have previously reported increased plasma levels of thrombin and APC in patients with non-COVID sepsis [17,18]. In the present study, both key enzymes of the PC system were assessed comparatively in COVID-19 and non-COVID sepsis using oligonucleotide-based enzyme capture assays (OECAs). As a reference for thrombin-induced APC formation in the absence of endothelial dysfunction, thrombin and APC were also measured in healthy volunteers, in whom limited and standardized coagulation activation was induced by recombinant activated coagulation factor VII (rFVIIa). The results provide direct evidence that the functionality of the thrombin–endothelium–APC axis is impaired in COVID-19 sepsis.

## 2. Materials and Methods

This prospective, observational cohort study was conducted from December 2015 to January 2023. Patients with COVID-19 were recruited between October 2020 and January 2021, during the second wave of COVID-19 in Germany, which started at the end of September 2020 and was still ongoing in March 2021 [19]. Patients with non-COVID sepsis were recruited between December 2015 and May 2016. Healthy subjects were recruited between July 2016 and January 2023. All study procedures were performed in compliance with relevant laws and institutional guidelines and have been approved by the Medical Ethics Commission II of the Faculty of Medicine Mannheim, University of Heidelberg (reference number 2015-526N-MA from 2015, covering the non-COVID sepsis cohort) and the Institutional Review Board and Ethics committee of the Medical Faculty of the University of Bonn (reference number 016/16 from 2016, covering the cohort of stimulated healthy subjects, and reference number 134/20 from 2020, covering the COVID-19 sepsis cohort). Written informed consent was received prior to inclusion from the participants or, in the case of the patients, their caring relatives.

### 2.1. Study Participant Enrollment and Eligibility Criteria

**Figure 1:**
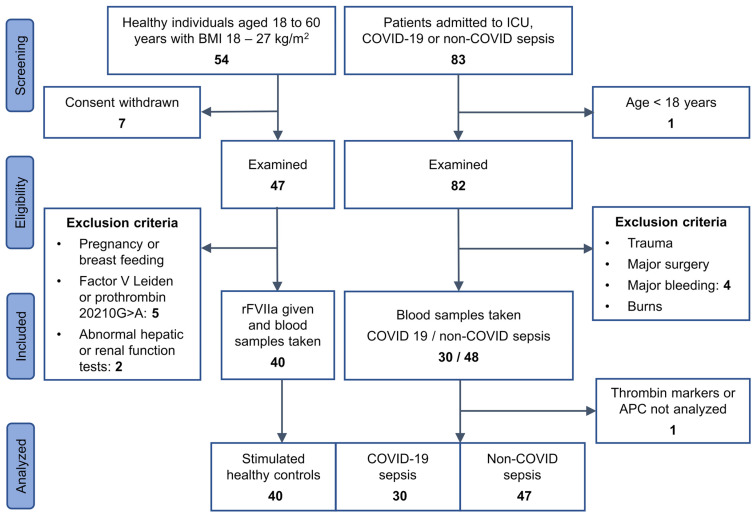
Study flow chart. Hepatic and renal function tests included transaminases, γ-glutamyl transferase, urea, and creatinine in serum. BMI, body mass index.

We included patients aged ≥ 18 years with COVID-19 sepsis (according to the Sepsis-3 definition) who had been admitted to the intensive care unit (ICU). The non-COVID cohort represented a subset of a bacterial sepsis cohort that had previously been published [17,18], and was defined according to the Sepsis-2 criteria [20]. Disease severity was determined by the sepsis-related organ failure assessment (SOFA) score. Diagnoses were confirmed by two ICU specialist physicians. In addition to age and sepsis as defined above, no inclusion criteria were specified for both patient cohorts. Exclusion criteria were trauma, major surgery, major bleeding, fibrinolytic therapy, or burns prior to admission to ICU, in order to minimize other factors affecting hemostasis. Healthy individuals were recruited from the blood donation service of the Institute of Experimental Hematology and Transfusion Medicine, University Hospital Bonn. They were tested for the factor V Leiden mutation and the prothrombin 20210G>A mutation using in-house methods. A diagram of study participant enrollment is shown in Figure 1, along with a description of additional selection criteria for healthy individuals.

### 2.2. Study Procedures

In patients with sepsis, blood samples were obtained within 24 h after admittance to the ICU. Patients were then followed up until death or discharge from the ICU. Healthy controls were required to fast overnight before morning administration of 15 µg/kg rFVIIa (NovoNordisk, Bagsværd, Denmark) as a single IV bolus injection and blood sampling after one hour. After discarding the first 2 mL, blood was drawn into citrate tubes (10.5 mmol/L final concentration, Sarstedt, Nümbrecht, Germany). Citrate tubes additionally contained argatroban (100 µmol/L final concentration, Mitsubishi Pharma, Düsseldorf, Germany) for thrombin measurement, as well as aprotinin (10 µmol/L, PanReac AppliChem ITW Reagents, Darmstadt, Germany) and bivalirudin (250 µg/mL, The Medicines Company, Oxfordshire, United Kingdom) for APC measurement. Plasma samples were obtained by centrifugation (2500× *g*, 15 min) within 30 min after blood draw and stored at less than −70 °C until assayed.

### 2.3. Laboratory Analysis of Blood Samples

The activity of the PC pathway was assessed by measuring plasma levels of APC, free thrombin, and the indirect thrombin markers prothrombin activation fragment 1+2 (F1+2) and thrombin-antithrombin complex (TAT). Thrombin and APC were measured using OECAs as originally described by Müller et al. [21,22]. Maxisorp Fluoronunc microtiter modules (Nunc A/S, Roskilde, Denmark) were initially coated with 10 µg/mL of bovine serum albumin-biotin (100 µL/well). After incubation at 4 °C overnight, wells were washed, and a solution of 10 µg/mL streptavidin was added. After incubation for 1 h at room temperature, wells were washed and blocked using 2% bovine serum albumin. Emptied plates were stored at −20 °C until used. For running the OECAs, 3′-biotinylated aptamers (HD1-22 for the thrombin-OECA or HS02-52G for the APC-OECA, both synthesized and purified from Microsynth, Balgach, Switzerland) were placed in the designated wells. After washing, the corresponding plasma samples were added to the wells. Samples for the APC-OECA were re-calcified before analysis by addition of 1 mol/L CaCl_2_, yielding a final concentration of 7.5 mmol/L. After incubation and washing, the fluorogenic peptide substrate I-1560 (Boc-Asp(OBzl)-Pro-Arg-AMC, Bachem, Weil am Rhein, Germany) or Pefafluor PCa (Pyroglu-Pro-Arg-AMC, Pentapharm, Basel, Switzerland), was added to the wells for detection of captured thrombin or APC, respectively. Changes in fluorescence over time were measured using a plate fluorescence reader (Synergy 2, BioTek Instruments, Bad Friedrichshall, Germany). Plasma-based calibration curves and two control samples were processed in parallel. Controls consisted of primed pooled normal plasma spiked with thrombin (CellSystems, St. Katharinen, Germany) or recombinant APC (Eli Lilly, Indianapolis, IN, USA) to achieve final concentrations of 5 and 0.5 ng/mL (136 and 13.6 pmol/L of thrombin, 91 and 9.1 pmol/L of rAPC). Calibrators covered a ½-log10 concentration range from 0 to 10 ng/mL of thrombin (0–272 pmol/L) or rAPC (0–182 pmol/L). Calibrator data were interpolated by a four-parameter curve fit and used to calculate the thrombin or APC concentration in the samples. All samples were assayed in triplicate, and aliquots of the same controls were used in all runs. If the measured plasma concentration of a control deviated more than 10% from the target analyte concentration, the run was repeated.

Concentrations of F1+2 and TAT were determined using the Enzygnost F1+2 (monoclonal) assay and the TAT micro assay, respectively (Siemens Healthcare Diagnostics Products, Marburg, Germany). Prothrombin time (PT), activated partial thromboplastin time (aPTT), and plasma levels of PC and D-dimer were determined using the BCS XP or Atellica Coag 360 system (Siemens Healthcare Diagnostics, Eschborn, Germany) and corresponding reagents.

### 2.4. Statistical Analysis

Data are generally presented as median and interquartile range (IQR). The normality of data was tested using the Shapiro–Wilk test. To compare continuous data between cohorts, the Mann–Whitney test or the Kruskal–Wallis test was used, with the latter being followed by pairwise correction using the Dunn procedure and Bonferroni correction for multiple testing. Frequency data were compared using a chi-square test or the Fisher’s exact test. Values of *p* ≤ 0.05 were considered statistically significant. Correlations were assessed using Pearson correlation coefficients. All calculations were performed using the XLSTAT statistical and data analysis solution software, version 2021.4.1.1214 (Addinsoft, Boston, MA, USA). Statistical analysis was performed by H.R. All authors have complete and ongoing access to primary data.

## 3. Results

### 3.1. Study Population Characteristics

The final study population included patients with sepsis due to COVID-19 (n = 30) or other infections (n = 47), as well as 40 healthy controls who completed administration of rFVIIa and follow-up blood sampling. Both patient populations were similar regarding the majority of assessed demographic characteristics (age and sex), preexisting comorbidities (diabetes, immunosuppression, malignant disease, end-stage renal failure), and disease severity as determined by the SOFA score (Table 1).

A higher proportion of patients in the COVID-19 cohort than in the non-COVID sepsis cohort passed away in the ICU (67% versus 38%, *p* = 0.015), required mechanical ventilation (93% versus 70%, *p* = 0.020), or received therapeutic anticoagulation with heparin (50% versus 0%, *p* = 4 × 10^−8^). With a value of 28.5 versus 0.5 µg/L, median levels of the sepsis marker procalcitonin were significantly higher in the non-COVID sepsis cohort than in the COVID-19 cohort (*p* = 1 × 10^−7^). The healthy control group differed from both patient cohorts with respect to sex and age, as it deliberately consisted of 50% males/females and did not include subjects of 60 years or older for safety reasons regarding rFVIIa administration. White blood cells (WBCs, median 6.1 × 10^9^/L) and platelet count (251 × 10^9^/L) lay within normal ranges in healthy controls.In healthy controls, WBCs were lower, and platelet count was higher than in the COVID-19 (9.9 × 10^9^/L, 133 × 10^9^/L) and non-COVID sepsis (11.6 × 10^9^/L, 181 × 10^9^/L) cohorts (*p* ≤ 3 × 10^−5^) (Table 1).

### 3.2. Routine Hemostasis Parameters

**Figure 2:**
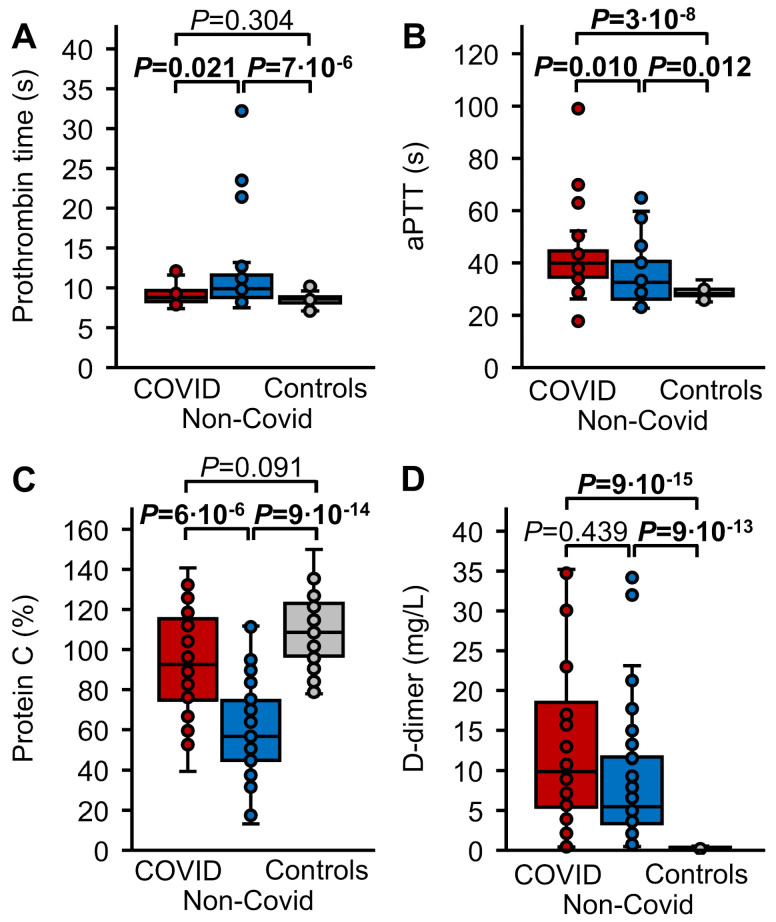
Hemostasis parameters. (**A**) PT in patients with COVID-19 induced sepsis (red, n = 30), non-COVID sepsis (blue, n = 37), and healthy controls (grey, n = 40). (**B**) aPTT in COVID-19 (n = 30) and non-COVID induced sepsis (n = 43), and healthy controls (n = 40). (**C**) Plasma levels of PC and (**D**) D-dimer in COVID-19 (n = 30) and non-COVID induced sepsis (n = 47), and in healthy controls one hour after IV administration of 15 µg/kg rFVIIa (n = 40). Data are presented as box plots indicating quartiles and median of the data, the whiskers extending up to 1.5 times the IQR from the box, and circles showing outlying values. *p* values were calculated using the Kruskal–Wallis test followed by pairwise comparison using the Dunn procedure. The Bonferroni method was used to correct for multiple comparisons (n = 3). Values of *p* ≤ 0.05 are shown in bold font.

The PT was significantly more prolonged (*p* ≤ 0.021) in the non-COVID sepsis cohort in comparison to healthy controls and to COVID-19 patients, at 9.9 (8.8–11.6) s versus 8.6 (8.1–8.8) s and 8.8 (8.3–9.6) s, respectively, but it did not differ between COVID-19 sepsis patients and healthy controls (Figure 2A). The aPTT was longer (*p* ≤ 0.012) in both patient groups than in healthy controls (28, 28–30 s), with 40 (35–45) s in the COVID-19 cohort and 33 (26–40) s in the non-COVID sepsis cohort, and longer in COVID-19 than in non-COVID sepsis patients (*p* = 0.010) (Figure 2B). Plasma levels of PC were decreased (*p* ≤ 6 × 10^−6^) in the non-COVID sepsis cohort (57, 45–75%) compared with healthy controls one hour after rFVIIa administration (109, 99–123%) and COVID-19 patients (93, 76–115%). They did not differ significantly statistically between the COVID-19 cohort and healthy controls (*p* = 0.091) (Figure 2C). With values of 9.88 (5.82–16.83) mg/L in COVID-19 patients and 5.47 (3.48–11.60) mg/L in non-COVID sepsis patients, D dimer levels in both patient cohorts were drastically increased (*p* ≤ 9 × 10^−13^) in comparison to stimulated healthy controls (0.26, <0.19–0.36 mg/L), but did not differ significantly between COVID-19 and non-COVID sepsis patients (*p* = 0.439) (Figure 2D).

### 3.3. Thrombin Markers and APC

**Figure 3:**
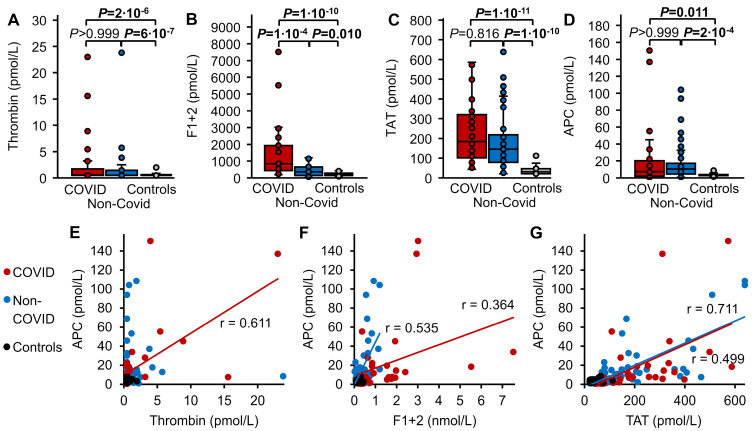
Thrombin markers and APC. Plasma levels of (**A**) thrombin, (**B**) F1+2, (**C**) TAT, and (**D**) APC were compared in patients with COVID-19 induced sepsis (red, n = 30), non-COVID sepsis (blue, n = 47), and in healthy controls one hour after IV administration of 15 µg/kg rFVIIa (grey, n = 40). Data are presented as box plots indicating quartiles and median of the data, the whiskers extending up to 1.5 times the IQR from the box, and circles showing outlying values. *p* values were calculated using the Kruskal–Wallis test followed by pairwise comparison using the Dunn procedure. The Bonferroni method was used to correct for multiple comparisons (n = 3). Values of *p* ≤ 0.05 are shown in bold font. The correlation between plasma levels of (**E**) thrombin, as well as plasma levels of the thrombin formation markers (**F**) F1+2 and (**G**) TAT, and plasma levels of APC. The Pearson correlation coefficient (r) is shown in cases of *p* ≤ 0.05.

Free thrombin, F1+2, TAT, and APC were significantly increased (*p* ≤ 0.011) in the plasma of both the COVID-19 and non-COVID sepsis cohorts compared to healthy controls stimulated with rFVIIa, with median thrombin levels of 0.54, 0.48, and < 0.46 pmol/L, F1+2 levels of 846, 360, and 209 pmol/L, TAT levels of 185, 146, and 31 pmol/L, and APC levels of 7.4, 10.5, and 3.1 pmol/L, respectively (Figure 3A–D). Among these parameters, only F1+2 levels were significantly higher in COVID-19 patients than in non-COVID patients (*p* = 1 × 10^−4^) (Figure 3B), whereas plasma levels of thrombin, TAT, and APC did not differ between both patient groups (Figure 3A,C,D). Thrombin and APC levels showed correlation only in the COVID-19 cohort (r = 0.611), not in the non-COVID or healthy control groups (Figure 3E). F1+2 and TAT levels were correlated with APC levels in COVID-19 (r = 0.364 and r = 0.499, respectively) and non-COVID patients (r = 0.535 and r = 0.711, respectively), but not in healthy controls (Figure 3F,G).

**Figure 4:**
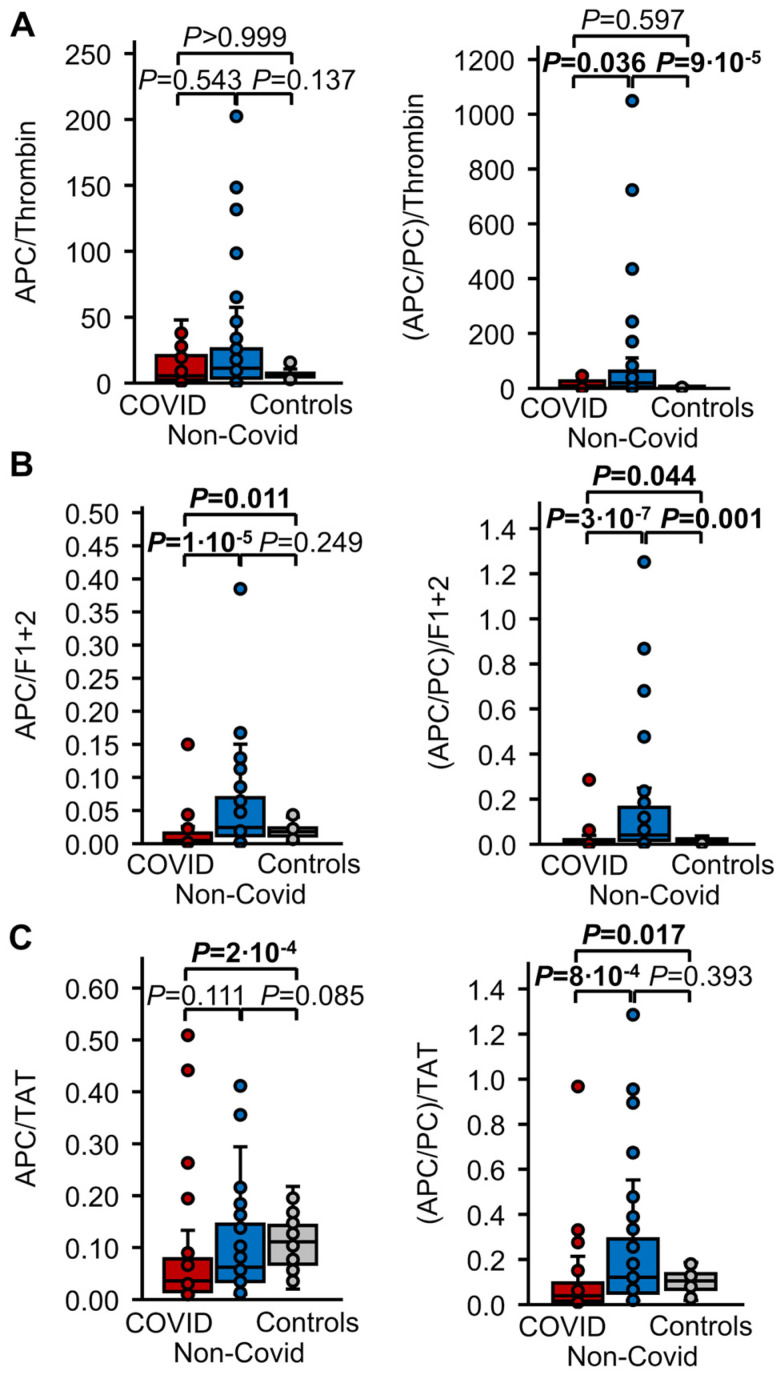
APC in plasma in relation to thrombin formation. Plasma levels of APC, and the ratio of APC/PC in relation to plasma levels of (**A**) thrombin, (**B**) F1+2, and (**C**) TAT were compared in patients with COVID-19 induced sepsis (red, n = 30), non-COVID sepsis (blue, n = 47), and in healthy controls one hour after IV administration of 15 µg/kg recombinant activated factor VII (grey, n = 40). Data are presented as box plots indicating quartiles and median of the data, the whiskers extending up to 1.5 times the IQR from the box, and circles showing outlying values. *p* values were calculated using the Kruskal–Wallis test followed by pairwise comparison using the Dunn procedure. The Bonferroni method was used to correct for multiple comparisons (n = 3).

To assess the relation between thrombin formation and APC generation in the three cohorts, the ratios between thrombin markers and APC were compared. To account for differences in plasma levels of PC as a potential influencing factor of thrombin-induced APC formation, we also compared the ratios of thrombin markers and plasma levels of APC/PC. These data are summarized as median and IQR in Table 2 and are shown in Figure 4.

The ratio of APC to thrombin did not differ statistically significantly between the three cohorts (Figure 4A), whereas the medians of APC/F1+2 and APC/TAT were approximately 4-fold and 3-fold lower, respectively, in the COVID-19 cohort than in stimulated healthy controls (*p* = 0.011 and *p* = 2 × 10^−4^, respectively). Additionally, the median of APC/F1+2 was approximately 5-fold lower in COVID-19 than in non-COVID sepsis patients (*p* = 1 × 10^−5^). APC/F1+2 and APC/TAT did not differ significantly between non-COVID sepsis patients and stimulated healthy controls (Table 2, Figure 4B,C). The ratios of all thrombin markers and APC/PC were significantly lower in the COVID-19 group than in the non-COVID group. The medians of (APC/PC)/thrombin and (APC/PC)/F1+2 were approximately 1.3 fold and 1.5-fold higher, respectively, in non-COVID patients than in stimulated healthy controls (*p* = 9 × 10^−5^ and *p* = 0.001, respectively). (APC/PC)/F1+2 and (APC/PC)/TAT, but not (APC/PC)/thrombin, were significantly lower in the COVID-19 cohort than in stimulated healthy controls (Table 2, Figure 4).

## 4. Discussion

In this study, patients with COVID-19 sepsis and non-COVID sepsis showed higher plasma levels of free thrombin, the indirect thrombin markers F1+2 and TAT, and APC than healthy controls after low-grade coagulation activation. Increased F1+2 levels were an especially prominent feature of COVID-19 sepsis, highlighting the extent of coagulation activation despite therapeutic anticoagulation in half of the cohort. We have previously studied the elevation of free thrombin and APC levels in non-COVID sepsis [17,18]. The indirect markers of thrombin formation, F1+2 and TAT, have been studied previously by others in critically ill patients with COVID-19 [7,23,24], but this study is, to the best of our knowledge, the first to investigate the activity level of the anticoagulant PC pathway in this patient group. The novelty of this study lies, first, in measuring the active hemostasis enzymes thrombin and APC in plasma, which has not yet been conducted in patients with COVID-19 and, second, in combining both parameters to assess the functionality of the PC pathway in these patients.

We have demonstrated previously in vivo that the functionality of the PC system can be assessed by comparative analysis of thrombin and APC formation [25]. We hypothesized that in COVID-19 sepsis and non-COVID sepsis the APC generating capacity of the endothelium could be reduced due to systemic endothelial damage, resulting in impaired APC formation in response to thrombin formation. Since APC plasma levels usually lie below quantifiable ranges in healthy individuals when the coagulation system is in a resting state [22], standardized thrombin and subsequent APC formation were induced by low-grade stimulation of the extrinsic coagulation by rFVIIa in the healthy control group, as described previously [25]. In contrast to both groups of sepsis patients, D-dimer levels remained within normal ranges in these stimulated healthy controls, indicating that thrombin formation was sufficiently counteracted to prevent fibrin formation.

Thrombin markers and APC were correlated in the non-COVID sepsis and COVID-19 sepsis cohorts, indicative of the dependency of PC activation on thrombin formation. They were not correlated in healthy controls, most probably because of the smaller spread of observed values. The ratio between APC levels and levels of the indirect thrombin markers F1+2 and TAT was significantly lower in patients with COVID-19 sepsis, but not in the non-COVID sepsis group, in comparison to healthy controls, suggesting relatively reduced APC formation in response to thrombin formation. When APC levels were normalized against PC activity in plasma, to focus on endothelial effects on the thrombin-APC axis, these differences prevailed. Furthermore, these normalized ratios were uniformly lower in the COVID-19 sepsis group than in the non-COVID sepsis group, indicating relatively less endothelial PC activation in the former than in the latter. Compared with the stimulated healthy controls, the normalized ratios between APC and F1+2 and between APC and TAT were higher in the non-COVID sepsis group, suggesting increased activity of the PC system. Taken together, these results strengthen previous evidence that dysregulation of the PC system contributes to the prothrombotic phenotype in COVID-19 associated coagulopathy [7,11,12,13,14,15,16].

The coagulopathy associated with COVID-19 involves interactions between the innate immune response, the coagulation and fibrinolytic pathway, and the vascular endothelium [11]. Understanding the contribution of the PC pathway to this complex pathomechanism is especially important, given its role in both antithrombotic and cytoprotective endothelial mechanisms [26]. Different mechanisms, or a combination thereof, are involved in endothelial damage, and may therefore be involved in a decreased APC response in COVID-19. In the early stages of the disease, SARS-CoV-2 infects type II pneumocytes, causing damage to the adjacent vascular endothelium through increased inflammation, infiltration of activated neutrophils, release of neutrophil extracellular traps (NETs), complement activation, and microvascular thrombosis [27,28]. Vascular leakage, induced via direct effects of the virus on its host cell surface receptor angiotensin-converting enzyme 2 (ACE2), further contributes to vascular endothelial injury [29]. Hyperinflammation and endothelial damage, which not only impairs the cellular receptors of the PC system, EPCR, and TM, but also tissue factor pathway inhibitor and fibrinolytic activity, contribute together to COVID-associated coagulopathy [30]. Against this background, the importance of anticoagulant and anti-inflammatory treatment in patients with COVID-19 becomes evident [31]. Moreover, one might speculate as to whether the cytoprotective effects of APC may be especially effective to counteract thrombotic complications in COVID-19, particularly as EPCR-dependent PAR signaling also induces anti-inflammatory effects [11].

Interestingly, the APC response to thrombin formation appeared to be unimpaired in non-COVID sepsis despite significantly reduced PC levels. This observation supports the results of the SCARLET trial, which failed to show a reduction in 28-day mortality with the use of recombinant TM in the treatment of sepsis-induced coagulopathy [32]. The PT in the COVID-19 sepsis group was comparable to the healthy controls; only the aPTT was prolonged due to therapeutic anticoagulation with heparin. Despite overall more deranged hemostasis parameters in the non-COVID sepsis group, as indicated by a prolonged PT, mortality was higher in the COVID-19 group. This could be partly due to the high incidence of fatal intracerebral hemorrhage in COVID-19 compared to non-COVID patients with therapeutic anticoagulation [6,33]. Other significant differences between both groups (procalcitonin levels and mechanical ventilation) were due to the differences in the host response to various pathogens. As both groups did not differ with regards to preexisting comorbidities and disease severity, it is difficult to explore if factors other than COVID-19 contributed to the decreased APC response. One major difference between groups that probably influenced thrombin generation and subsequent APC response was therapeutic anticoagulation with heparin in the COVID-19 cohort. One can assume that without heparin, thrombin formation rates and subsequent APC formation rates would have been higher. To rule out such an effect, we assessed the APC response in relation to thrombin and indirect thrombin markers, respectively.

The main limitations of this study are the relatively small sample size, the cross-sectional nature of the study, and the fact that the stimulation approach in healthy volunteers is not an ideal model of coagulation activation in sepsis. However, this is the first study investigating thrombin-induced APC formation in COVID-19 sepsis; thus, the obtained results should be considered hypothesis-generating. Further limitations included the collection of samples in different centers and over a long study period, and that we did not measure other parameters indicative for endothelial dysfunction such as von Willebrand factor, soluble TM, or soluble EPCR. Another limitation is that all samples from patients with COVID-19 sepsis were collected during the second wave of COVID-19, thus limiting conclusions regarding later waves and variant strains. To control for potential sources of bias or imprecision, measurement of parameters was conducted following ISO standards, except for the OECAs for thrombin and APC measurement, whose performance has been extensively studied previously [23,24]

## 5. Conclusions

Our results support previous findings showing different phenotypes of coagulopathies in COVID-19 sepsis and non-COVID sepsis. In COVID-19 sepsis, the prothrombotic phenotype dominates, characterized by a relatively reduced APC response to thrombin generation, which could be explained by endothelial dysfunction. In contrast, concurrence of both prothrombotic and pro-hemorrhagic changes represent the dominant phenotype in non-COVID sepsis. Further studies combining the measurement of thrombin markers and APC with direct assessment of endothelial cell dysfunction in patients with different causes of sepsis are needed to investigate this hypothesis.

## Figures and Tables

**Table 1:** Characteristics of the study population.

	COVID-19 (n = 30)	Non-COVID	Stimulated Healthy Controls (n = 40)	*p* *
Age in years	63 (59–66)	63 (52–71)	29 (27–48)	0.54 (**3 × 10^−12^**)
Male sex	26 (87%)	38 (81%)	20 (50%)	0.553 (**0.007**)
Deceased	20 (67%)	18 (38%)	-	**0.015**
Diabetes	11 (37%)	14 (30%)	-	0.527
Immunosuppression	3 (10%)	7 (15%)	-	0.732
Malignant disease	2 (7%)	12 (26%)	-	0.066
End-stage renal failure	1 (3%)	5 (11%)	-	0.395
Mechanical ventilation	28 (93%) ^†^	33 (70%)	-	**0.020**
Therapeutic heparin	15 (50%)	0	-	**4 × 10^−8^**
SOFA score	13 (11–13)	12 (11–14)	-	0.932
Procalcitonin	0.5 (0.4–1.1)	28.5 (1.6–50.0) ^‡^	-	**1 × 10^−7^**
CRP (mg/L)	186 (114–240) ^§^	207 (125–298)	-	0.376
WBCs (10^9^/L)	9.9 (8.0–16.0)	11.6 (5.6–20.5)	6.1 (4.9–6.8)	0.534 (**3 × 10^−6^**)
Platelets (10^9^/L)	133 (91–184)	181 (115–269)	251 (215–287)	0.204 (**3 × 10^−5^**)

Dichotomous data are presented as counts and percentages and continuous data are summarized as medians and interquartile ranges. *p* values refer to differences between the cohorts with COVID-19 induced and non-COVID induced sepsis and were calculated using the Mann–Whitney test for continuous data, the chi-square test for frequency data, and the Fisher’s exact test for cell frequencies below 5. * *p* values in brackets refer to differences between the three cohorts (i.e., including the cohort of stimulated healthy controls, where applicable) and were calculated using the Kruskal–Wallis test for continuous data and the Fisher’s exact test for male sex. Values of *p* ≤ 0.05 are shown in bold font. ^†^ Thereof 23 on extracorporeal membrane oxygenation. ^‡^ n = 24. ^§^ n = 23. CRP, C-reactive protein; WBCs, white blood cells.

**Table 2:** APC in relation to thrombin markers.

	COVID-19 (n = 30)	Non-COVID (n = 47)	Stimulated Healthy Controls (n = 40)
APC/Thrombin	5.5 (2.7–18.7)	11.3 (3.9–25.4)	data
APC/F1+2	**0.005 (0.003–0.014)**	0.024 (0.012–0.068)	0.018 (0.012–0.023)
APC/TAT	**0.04 (0.02–0.07)**	0.06 (0.03–0.14)	0.11 (0.07–0.14)
APC/PC (pmol/L)	**6.7 (2.4–23.0)** *	**15.3 (5.9–39.1)** ^†^	3.0 (2.2–4.0)
(APC/PC)/Thrombin	9.0 (3.4–24.0)	**19.4 (5.9–59.0)**	5.1 (4.4–7.2)
(APC/PC)/F1+2	**0.007 (0.003–0.018)**	**0.041 (0.017–0.151)**	0.016 (0.012–0.024)
(APC/PC)/TAT	**0.04 (0.02–0.09)**	0.12 (0.05–0.28)	0.11 (0.07–0.14)

Data are presented as median and interquartile ranges. Differences between cohorts were assessed using the Kruskal–Wallis test followed by pairwise comparison using the Dunn procedure. The Bonferroni method was used to correct for multiple comparisons (n = 3). Statistically significant differences (*p* ≤ 0.05) to stimulated healthy controls are indicated in bold font. Statistically significant differences between the COVID-19 and non-COVID sepsis cohorts are underlined. * versus stimulated healthy controls *p* = 0.005, versus non-COVID sepsis *p* = 0.260. ^†^ versus healthy controls *p* = 1.9 × 10^−7^. All other *p* values are provided in Figure 4.

## Data Availability

The data presented in this study are available on request from the corresponding author due to privacy and ethical restrictions.
